# Health systems collaboration can strengthen climate change resilience: Insights from Indigenous knowledges in the Latin American region

**DOI:** 10.1371/journal.pgph.0005958

**Published:** 2026-04-08

**Authors:** Carol Zavaleta-Cortijo

**Affiliations:** 1 Intercultural Citizenship and Indigenous Health Unit (UCISI), School of Public Health and Administration (FASPA), Cayetano Heredia Peruvian University (UPCH), San Martín de Porres, Lima, Perú; 2 Indigenous Peoples Observatory Network (IPON), Cayetano Heredia Peruvian University (UPCH), San Martín de Porres, Lima, Perú; Washington State University, UNITED STATES OF AMERICA

## Abstract

Strengthening the resilience of health systems is a recognised pathway for responding to the impacts of climate change. However, current approaches often rely on biomedical models that exclude Indigenous Peoples and their knowledges. In Latin America, Indigenous communities have long maintained comprehensive health systems that support both individual and collective well-being, rooted in traditional medical knowledges and close engagement with biodiversity. These systems are grounded in lived experience and provide valuable evidence for addressing climate-related health risks. This essay explores how collaborative approaches rooted in Indigenous health practices can enhance climate resilience in the Latin American region. Drawing on peer-reviewed literature and personal experience in the Andean and Amazonian regions, I argue for health system responses grounded in Indigenous leadership, knowledges and health needs to offer context-specific and high-quality care to adapt to and mitigate climate change. In doing so, this approach can advance health and climate policies by ensuring that justice, meaningful participation, and Indigenous Peoples ‘ health are not left behind as in the past.

## 1. Background

Strengthening health systems’ resilience to climate change is recognised as one critical pathway to adapting to and mitigating the impacts of climate change worldwide [1]. However, most of the actions and methodologies to understand and respond to climate impacts are being implemented following biomedical approaches, with little participation of Indigenous Peoples in this process [2]. Indigenous participation is critical for methodological reasons, but more importantly, to tackle the exclusion and discrimination that Indigenous Peoples have experienced within the health and medical sciences globally, including in the Latin American region [3,4]. Indigenous Peoples’ participation includes the recognition and attention to Indigenous knowledges in framing vulnerabilities and resilience to climate change impacts on health and health systems [5]. Health systems’ resilience is inherently context-specific [6], implying that Indigenous Peoples’ lived experiences with changing climatic conditions must be part of any response or research that involves their health; otherwise, the knowledge would be incomplete or, even worse, recommendations could create unexpected consequences and perpetuate inequities [7–9].

**Fig 1:**
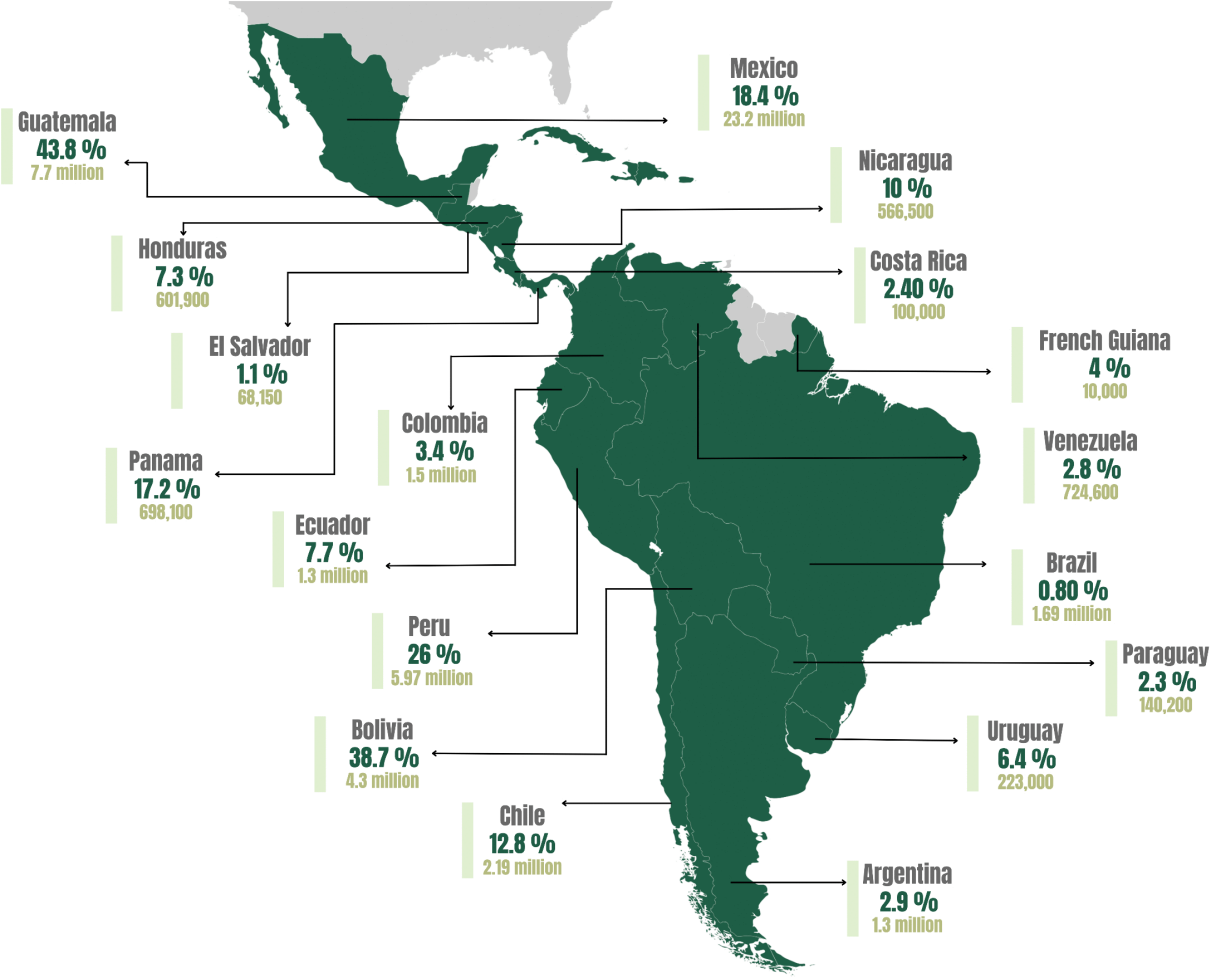
The map shows the percentage of the total population of Indigenous Peoples in each country in the Latin American region. Source [11] and data in supplementary materials A. Mapa created by Andrea Valdivia-Gago.

In Latin America region, Indigenous Peoples have practised their own medicine and traditional healing practices for millennia. Their survival is reflected in more than 700 Indigenous Peoples speaking 560 different Indigenous languages, comprising 8% of the total population [10]. Fig 1 shows the population of Indigenous peoples in the Latin American region, with data included in S1 Table.

Indigenous health systems, rooted in Indigenous knowledges, their health needs, and the use of biodiversity, passed down through generations, form a healing system designed to maintain and sustain individual and collective well-being. There are references in the literature about Indigenous Peoples in the Andeans using techniques and resources from biodiversity to solve health conditions [12,13], but more importantly to me is that it is not possible to imagine that Indigenous societies did not have a system to cure their people and have survived until today. My own family in the Peruvian coast, for example, used herbal preparations to alleviate menstrual pain and implemented therapeutic processes to address imbalanced health conditions caused by coping with different “shocks,” such as being frightened by a large animal or experiencing extreme flooding. Later, while working with Indigenous communities in the Amazon, I learned that Indigenous Peoples do not simply have a few practices, but rather a comprehensive and interconnected system of practices and norms based on specialised knowledge of local biodiversity that is transmitted within communities and families [14,15].

The challenge now is how to translate Indigenous knowledges into policies that can support action in the climate change crisis. A collaborative approach between Indigenous health systems and biomedical understandings of health opens opportunities to foster climate-transformative leadership and to reimagine health systems’ resilience, to better serve peoples in the Latin American region, including Indigenous Populations, who are among the most at risk, yet have contributed the least to global warming [16]. To spark this collaborative approach, efforts should be made to revitalise evidence and experiences generated by Indigenous Peoples and make them more accessible to a broader audience, especially within the public health community.

## 2. Objective

This essay introduces a series of concepts related to health and health systems to support a collaborative approach that strengthens health system resilience to climate change by restoring and uplifting Indigenous health practices to inform policies in the Latin American region. In doing so, I aim to contribute to the research and academic community in public health, including Indigenous and non-Indigenous leaders interested in exploring health risks related to climate change from a health systems perspective.

## 3. Methods

### 3.1. Health systems resilience

I recognise that there are multiple definitions of resilience, including those that focus on returning to an original state and others that support transformation [17]. For this essay, I use the definition proposed by the World Health Organization (WHO): “*A climate-resilient health system is one that is capable of anticipating, responding to, coping with, recovering from and adapting to climate-related shocks and stress, so as to bring sustained improvements in population health despite an unstable climate.*” [1]This definition acknowledges that health systems must not only adapt but may also need to transform to maintain health [2].

Part of this transformation involves recognising that health systems cannot be separated from the environment, the cultures and sentiments of the people they serve [18,19] It also requires accepting, with humility, that public health systems and tools are insufficient to respond to the immediate and long-term consequences of climate change [20]. Building climate-resilient health systems, therefore, demands a broader understanding of the capacities needed to anticipate, adapt to, and recover from climate shocks. In this context, there is an opportunity to recognise Indigenous knowledges—already present within many Latin American health systems, even if often rendered invisible—and to work collaboratively across health systems to strengthen their resilience.

While I draw on literature from across Latin America, this essay is grounded in my professional experience in the Peruvian Amazon, family memories from the Peruvian Andeans, and my last four years living in Bolivia, where I have also met with Indigenous health practitioners. It is important to remember that Indigenous knowledges are still largely transmitted orally, which is why I consider using my personal experiences, combined with peer-review literature, to be valid.

I begin by examining how Indigenous Peoples and their health-related practices remain unrecognised despite the important contributions they make to health system strengthening. In the second section, I reflect on how Indigenous understandings of health align with the World Health Organisation’s six building blocks of health systems [1,21]. I begin by presenting how Indigenous knowledges conceptualise health and disease, and then explore more deeply how these sociocultural practices intersect with the building blocks, aiming to ilustrate possible parallels among knowledge systems. Finally, I discuss how climate change is likely to impact Indigenous health beyond typical morbidity and mortality indicators. I conclude by situating these reflections within the broader climate change context in Latin America, to emphasise how meaningful collaboration can emerge to inform climate policies.

## 4. Indigenous peoples’ knowledges and health needs

### 4.1. Indigenous peoples and discriminatory practices in the Latin American region

The disconnect between Indigenous and non-Indigenous health practices in Latin America has made the action to reduce global warming and prepare health systems to adapt to climate change unrealistic. In Latin America, discriminatory practices in formal health systems are still prevalent and have led Indigenous patients to avoid seeking health care [4,22–26]. Although countries like Peru, Bolivia and Chile have adopted intercultural health policies [27–29], discriminatory practices remain, particularly a lack of acceptance and collaboration with traditional healers [30]. Discriminatory practices and a lack of understanding of Indigenous health systems and traditional knowledge mean that Indigenous Peoples will continue to be excluded from access to high-quality health care [31], compounding their climate change health risks and perpetuating historical global injustices [32]. For instance, during the COVID-19 pandemic, Wayuu families in Colombia and Venezuela struggled to access culturally adapted health care services in a context where food insecurity remained highly prevalent [8]. These systemic conditions aggravate the impacts of climate change-induced disruptions to regular seasonal patterns, undermining food and water security among the Wayuu Indigenous Peoples [33].

At the same time, formal health systems often ignore the contribution that Indigenous Peoples and their knowledge systems have made to support the health of all types of life on our planet. In other regions of the world, health authorities have reported that Indigenous traditional and ecological knowledge has favoured positive health outcomes in facing climate change [34,35]. For example, engaging in traditional activities and maintaining strong community ties have been found to be protective factors that enhance the mental health of Indigenous youth in the Circumpolar region [36]. In the Peruvian Amazon, it was found that Shawi and Ashaninka Indigenous Peoples use, protect and sustain the forest as a source of medicinal and preventive health measures in anticipation of climate variability and in response to extreme climate events [37]. As I mentioned in the background section, and reported from Mexico [38], medicine for women’s health is another contribution of Indigenous Peoples to health systems in Latin America.

Indeed, discriminatory practices and poor-quality health care have been identified as key shortcomings of health systems that were still unable to adapt to new evidence and changing people’s needs [39]. Indigenous knowledges offer entry points and evidence to improve multiple health outcomes like women´s health, mental health and nutrition, and support the rights to health, food, and cultural identity within a health systems model that urgently needs to adapt to the emerging climate crisis.

### 4.2. Indigenous perspectives on health and the WHO´s building blocks

#### 4.2.1. Health and disease.

**Fig 2:**
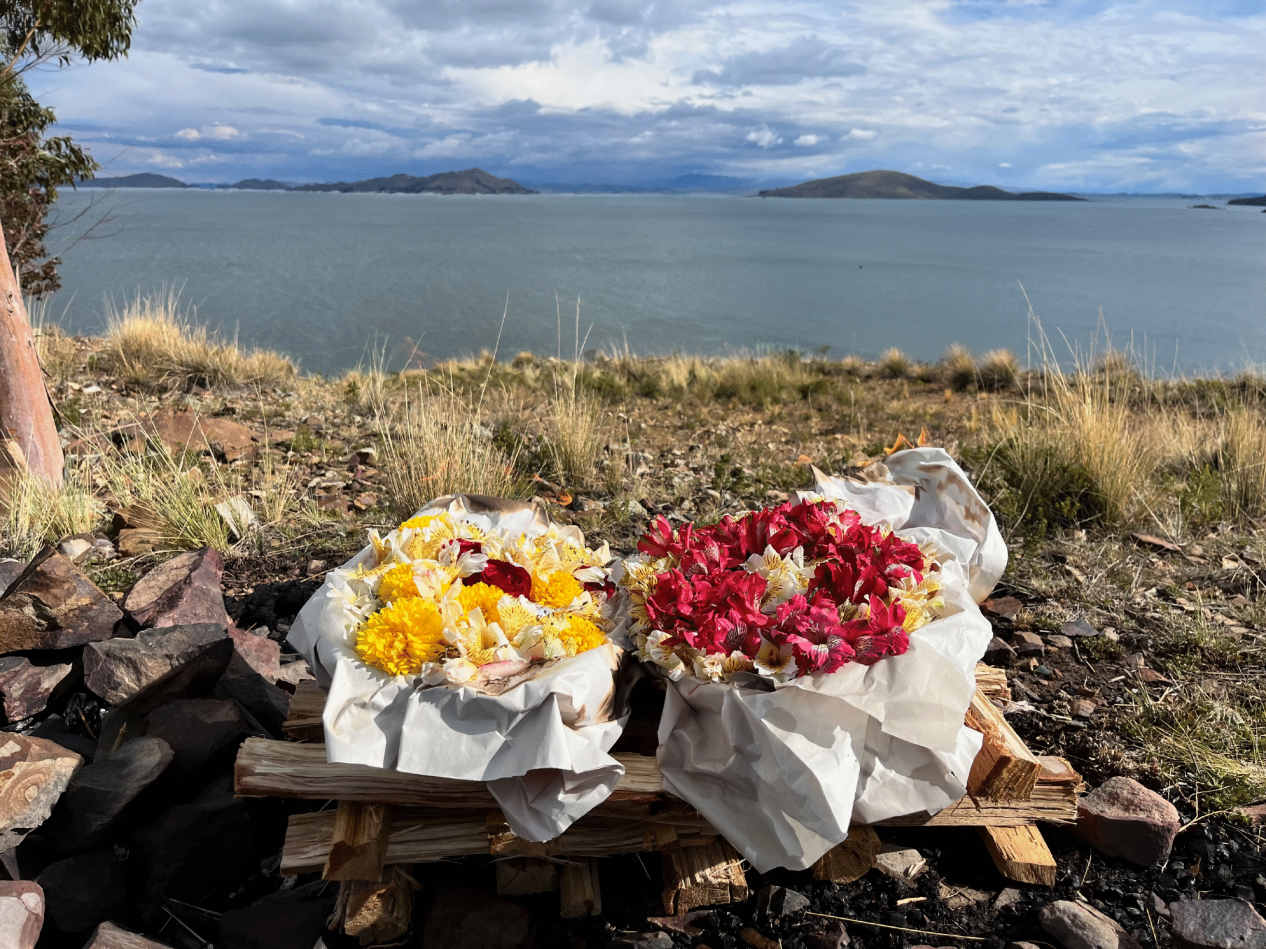
A traditional Bolivian healer helped me make this offering to Lake Titicaca, as a gesture of gratitude to my female ancestors (Feb 1^st^ 2024).

Indigenous perspectives on health recognise that maintaining physical, spiritual, emotional and collective well-being is crucial in guaranteeing healthy people on a healthy planet [40,41]. Collective well-being refers to the importance of being happy with family and community [15,42]. Hunting with relatives, sharing food with community members, and taking care of home gardens collectively with family members are a few examples for collective well-being [43]. Indigenous Peoples in the Peruvian Amazon have described that health is “having good foods”, “having tranquility”, “living in harmony [in the community]”, “living with non-concerns” [40]. This means that unbalanced conditions between natural and social systems cause negative impacts for individuals, families and communities. This unbalanced condition indicates that the aetiology, or the pathophysiological explanation for diseases, is different when working with Indigenous Peoples. The source of disease under Indigenous understanding includes not only the state of the individual´s health, but also the relationship with the community and the environment. Therefore, in completing a comprehensive diagnostic and treatment healthcare plan, incorporating other dimensions like the relationships with relatives, community members and other entities that govern the health and behaviours of the land, rivers, and forests are all-important. I share a photo (Fig 2) from a well-being moment when I was introduced to Lake Titicaca in Bolivia. This experience exemplifies how Indigenous health practices embed well-being in the relationships between people and the natural world—relationships that climate policies must acknowledge to strengthen health system resilience.

#### 4.2.2. Health systems and Indigenous peoples.

Many Indigenous scholars and leaders are contributing to a growing movement calling for transformations in how health systems are conceptualised [44,45]. First, the understanding of health is expanding to include Indigenous knowledge systems in which caring for people, land, water, and territory is central [46]. As seen during the pandemic and during extreme climatic events, Shawi, Ashaninka, and Shipibo Peoples in the Amazon region turned to their ancestral knowledge to respond to large-scale health threats [22,47]. Second, health is increasingly recognised as deeply connected to the wellbeing of the natural world—Mother Earth, the rivers, the forests, Father Sun, and other entities that sustain life [46]. In the Brazilian Amazon, for example, food insecurity is being linked to the erosion of human and more-than-human relations, where the health of the river is intertwined with cultural preservation and the protection of sacred sites [48].

This perspective invites climate-resilient health responses that consider not only “*green solutions*,” but the broader ecological and spiritual systems that enable health and wellbeing. Learning from Indigenous knowledge systems, therefore, includes understanding their philosophical ways of knowing, being, and *envisioning* nature, illustrated, for example, by Indigenous philosophers in Bolivia who describe *andar con* or “*walking with”* as a way of knowing how to live in their territory [49]. From here, I begin to see where points of connection can emerge between Indigenous perspectives and the WHO global health systems approach.

#### 4.2.3. Indigenous peoples’ knowledges inform health systems building blocks.

Indigenous healthcare systems have common characteristics with the current health system approach proposed by the WHO, based on six building blocks. I will develop this section with the intention of providing a first look at how the two health systems, Indigenous and non-Indigenous, can eventually form a pathway for collaboration. By no means is this a strict review of the literature. Most importantly, it will require deep intercultural dialogue with Indigenous health practitioners and leaders to accurately reflect Indigenous systems of care for people, animals and other beings in our planet [49].

1. **Essential medical products and technologies** for Indigenous Peoples involve using natural resources and access to knowledge of how to use numerous species of flora and fauna, including strict diets [38,40,50]. The combination of diets, the knowledge to process specific plants, and unique materials to perform rituals are all “essential medical products” to deliver service to patients [40].
2. **Service delivery** involves treatments that include completing powerful rituals and using Indigenous knowledge. For example, it has been described that health specialists acquire therapeutic knowledge working under the mentoring of another Indigenous health specialist or through a gift from Mother Nature (e.g., visions and dreams) [50,51]. In addition, the knowledge transmission between mentor and mentee, or between the health specialist and the patient, is described as a sacred space, usually kept secret [51]. Secrecy was also reported to be associated with the rejection of dominant societies to recognise the value and the self-determination of using Indigenous medicines [50]. Service delivery is therefore possible when the Indigenous specialist is ready to perform the treatment.
3. **Health workforce has** a first level of care attention that is completed at home; mothers usually know plants to treat common health conditions affecting children, and this knowledge is transmitted from parents to children over generations within each extended family [52]. Women are generally the ones who are responsible for keeping this knowledge [50]. Indigenous health systems have their medical specialists; in countries like Peru, they are called *medicos, shamans, curanderos* [37]*,* in Bolivia *Yatiris,* and in Ecuador *Yachakuna* and *pajuyukuna* [51]. These specialists have the skills and knowledge to create therapeutic plans to restore the balance between the natural and social systems to prevent, protect and restore human health.
4. **The health information system** is based on the intergenerational transmission of knowledge about past events or “crises” that affected individual and community health, from parents and grandparents to Youth and children [47]. As part of the broader Indigenous movement, Indigenous Organisations in Latin America have implemented health programs using intercultural processes to transmit and elevate Indigenous practices and have actively responded during the COVID-19 pandemic [53]. Central to the communication health systems is Indigenous languages [54]. Given the diversity of spoken languages in Latin America, working closely with knowledge holders to strengthen health systems capabilities seems a priority, especially for risk communication.
5. **Leadership and governance** is perform by local authorities, they can be called *Apu* or *Chief* and also a *lider* [53]. In some communities, the local governance system involves many authorities, including the chief, the lieutenant governor, and the municipal agent [55]. Local authorities are responsible for protecting the entire community’s health, including overseeing Indigenous rights to access to the land and natural resources. They guide health, healing, and well-being decisions, often drawing from spiritual, ecological, and cultural wisdom. Their role is rooted in trust, local legitimacy, and intergenerational knowledge transmission.
6. **Health financing**, identifying a parallel related to financial resources to support Indigenous healthcare systems is challenging, especially in regions where access to monetary economies is limited. The use of money as a means of exchange for goods and services was introduced to Latin America during colonisation, five hundred years ago. Despite many policies aimed at improving income and employment opportunities for all, limited access to sufficient income and stable jobs remains a widespread issue, affecting Indigenous Peoples in particular, and reflected in the high levels of “*poverty*” reported among Indigenous families in Latin America [56]. In the case of Indigenous health care, it will determine the success of the treatment that is not necessarily associated with a monetary cost. Indigenous societies in many parts of the world rely on societal collective values like reciprocity, complementarity, and responsibility to ensure the health of the planet and peoples are preserved [44,57]; thus, successful treatment is equally related to conserving those core values than with the monetary aspects. However, in terms of financial costs, in Perú, it was reported that being treated by an Indigenous specialist could be more expensive than visiting official health facilities. Patients did not see this as a barrier, since the current in-place health services were perceived as not efficient or discriminatory (e.g., does not speak the Indigenous language) [31,50].

Understanding how Indigenous knowledges align with the WHO´s health system building blocks also provides a foundation for interpreting climate impacts to inform policies. Many Indigenous medical practices, including governance systems, the use of biodiversity for therapeutic purposes, and information systems rooted in diverse Indigenous languages, are being directly impacted by climate variability as well as broader socioeconomic and structural changes [58,59]. For this reason, health system resilience, from an Indigenous perspective, cannot be separated from the relationships between people and their culture. Recognising these linkages can guide policies toward responses built on intercultural dialogue across systems, rather than assimilation or hierarchy.

### 4.3. Indigenous perspectives on health and climate change impacts

In addition to the morbidity and mortality that can be created by climate variability and extreme events, conceptualizations of health also shape how Indigenous Peoples express symptoms and manifest their health needs and well-being [60]. For example, among the Achuar Indigenous Peoples, if a father hunts a bird that is not meant to be eaten, the baby may develop diarrhea [61], or Shawi men experience a feeling of guilt when they witness how animals in the forest are being reduced in numbers, affecting their ability to feed their family [62]. Rapidly changing climatic conditions affecting flora and fauna [56] can also accelerate the loss of social roles that Indigenous parents have to support their children, creating new stress on nuclear and extended families [63]. Furthermore, increasingly unpredictable seasons and extreme floods and droughts, compromise the availability and access to medicinal plants and water sources [52,64].

These examples show how Indigenous knowledge links health to the natural environment, in a relationship reflected in sociocultural practices. From this perspective, the impacts of climate change on Indigenous health are not limited to the presence of disease but also include disruptions in access to biodiversity and in relating with others. To better represent these interlinkages, a new set of policy indicators is likely needed [65], for example, measuring the health impacts of climate-driven losses of root and tuber species domesticated in Latin America and their connection to malnutrition in all its forms. Tubers and roots have been used by Indigenous peoples as part of their food systems for centuries, with scientists highlighting their nutritional and health importance [66,67], although evidence to capture their benefits for climate change adaptation and mitigation, as well as potential losses from an Indigenous health perspective, remains scarce. Given the persistent high prevalence of malnutrition in Latin America [68], a sole focus on biomedical interventions to improve access to nutritional services is insufficient and may overlook the contributions that Indigenous territories and food species offer to enhance health systems’ resilience.

Considering the quality of Indigenous health systems, I argue that their effectiveness can be understood as being adapted to their own ecosystems and responsive to the conditions in which people live. While biomedical systems offer remarkable therapeutic and preventive solutions like vaccination programs, Indigenous medical practices contribute other forms of care that are highly relevant for climate-related stressors. Through my early work in Amazonian communities [69,70] and my own family’s experience living in the Peruvian Andes, I developed a different understanding of what “isolation” means. In both regions, living far from urban centres is part of everyday life and reflects relationships with the territory, where practices like farming or hunting require specific weather patterns, types of soil and other ecosystem conditions. Distance is therefore not a sign of isolation, but a different way of living. When health systems fully see these practices as part of normal life, more realistic and culturally respectful policies can emerge. Our prior work has shown, for example, that biomedical technologies designed without consideration of environmental and cultural contexts may not function effectively in Indigenous settings [71]. Similarly, climate interventions could prioritise pain-relief options and hydration recommendations for Indigenous farmers who frequently experience musculoskeletal pain and headaches in the Peruvian Amazon [72]. Such forms of support reflect an approach that aligns more closely with Indigenous understandings of well-being and health priorities. In this way, collaboration does not replace biomedical care nor assume equivalence between systems. Instead, each system can strengthen the other when they work together, improving the quality and accessibility of health care to respond to climate change impacts.

### 4.4. Compounding environmental threats and implications for policy

Furthermore, there is evidence on unresolved conflicts related to economic activities involving the natural environment (e.g., illegal loggers, large-scale deforestation, oil companies), contributing to exposure to environmental health risks and related to greenhouse gas emissions [40,42,52,73], implying that not only climate change, but other stress are altering the health of Indigenous peoples, and thus, health policies need to be prepared for addressing compounding climate-related health risks. A better understanding and prioritisation of Indigenous knowledges and their health needs within health policies in Latin America can generate co-benefits by reducing ongoing health environmental threats and inform climate change mitigation efforts (e.g., reducing the use of fossil fuels) in the context of accelerating global warming.

## 5. Current climate trends and collaborative responses

### 5.1. Opportunities for collaboration

In the Amazon and Andean regions of Latin America, temperatures have already increased during the last four decades, with models concurring for the future, on a rise between 3°C and 5 °C above present-day levels, accompanied by more frequent extreme heat events, more severe droughts, and increased extreme flooding [74,75]. Heat and cold stress, waterborne diseases, vector-borne diseases, malnutrition in all its forms, and food sovereignty are all climate-sensitive health outcomes that Latin American health systems must address in the coming decades. Given the current trends in warming conditions and changes in rainfall patterns in Latin America, collaboration between Indigenous and non-Indigenous health systems represents a critical adaptation strategy to strengthen health systems by learning from ancestral wisdom and practices that have survived colonisation and globalisation [76].

A collaborative approach to designing and implementing health policies has been recommended by researchers and international organisations working in the Latin American region in maternal health and women’s health, and similar efforts can be mobilised to address climate change-related health risks [38,53,77]. In Colombia, a new national dietary guideline was developed through dialogues with Indigenous and other cultural communities [78]. By partnering with ancestral food knowledge and traditional culinary practices, this process shows how biomedical nutrition approaches can work alongside Indigenous knowledge holders in meaningful ways. In Nicaragua, efforts to improve health systems in the North Atlantic Autonomous Region have included partnerships between biomedical providers and Miskitu traditional healers [79]. Community organisations and universities facilitate intercultural workshops in which both systems treat patients, exchange knowledge, and coordinate care. Although these collaborations remain uneven and limited by funding and authority imbalances, they demonstrate that respectful processes can improve trust, community engagement, and access to care. Importantly, collaborative approaches should actively challenge discriminatory practices and revitalise Indigenous leadership and knowledges to effectively address Indigenous health needs and priorities [29,53,80].

### 5.2. Barries to collaboration

Despite these opportunities, implementing collaborative approaches remains challenging. Structural barriers—including limited recognition of Indigenous authority, and rigid biomedical protocols—continue to restrict meaningful engagement. Epistemic barriers are equally significant: Indigenous concepts of health, spirituality, and relationships with land are often misunderstood or dismissed, creating tensions that limit dialogue [46]. These challenges do not diminish the value of collaboration; rather, they highlight the need for careful, sustained processes that honour Indigenous autonomy (for example the autonomy to name their own health experts) and ensure well-supported spaces exist where different knowledge systems can meet without hierarchy.

Climate change itself represents a major barrier, as it threatens the very foundation of many Indigenous medical practices in Latin America by undermining biodiversity. Indigenous ways of living also link biodiversity to health resilience as part of their cultural practices [81]. The practices of *curanderos* in the Amazon and in the Andeans, for instance, help prevent the erosion and exploitation of ecosystems, reinforcing a key dimension of governance for adapting to climate change [82,83]. Although climate change threatens biodiversity and is often framed primarily as a source of vulnerability [84,85], respectful dialogue and care-centred collaboration with Indigenous knowledge holders, who understand the uses and meanings of biodiversity, offers a pathway to solutions and to building transformative resilience [86].

Of course, rebuilding trust is an essential step in bringing health systems together in Latin America, so that Indigenous practices—often considered secret and sacred—can be respectfully engaged with as valid therapeutic practices. Rebuilding trust begins by acknowledging the historical discrimination that Indigenous traditional healers have faced in many Latin American societies [30,87]. One way forward is to support the intergenerational transmission of knowledge and languages, so that young Indigenous people can openly learn and use the medicines and teachings of their grandparents.

## 6. Conclusion

The climate change resilience of health systems in the Latin American region lies in the collaboration among its peoples and their knowledges. A necessary first step is to address the persistent discrimination that Indigenous Peoples and their healing practices continue to face within official health systems, a change that should be translated into high-quality health care services for them. One step forward is the willingness of health care practitioners and educators to learn from Indigenous concepts about health. For the climate change era, Indigenous knowledges and perspectives should be the stones for designing, supporting, and implementing policies to adapt to and mitigate climate change, ensuring that justice, meaningful participation, and Indigenous leadership are not left behind as in the past.

## Supporting information

S1 Table Indigenous Peoples in Latin America: 2014 vs latest available data (up to 2025). (DOCX)
