# Supplementary material for: Health systems collaboration can strengthen climate change resilience: Insights from Indigenous knowledges in the Latin American region
Source: PLOS Glob Public Health. 2026 Apr 8;6(4):e0005958. doi: 10.1371/journal.pgph.0005958 (PMC13061210; doi:10.1371/journal.pgph.0005958)
Supplement: S1 Table — (DOCX) [file pgph.0005958.s001.docx]

**S1 Table. Indigenous Peoples in Latin America: 2014 vs latest available data (up to 2025)**

| **Country** | **Indigenous Peoples in 2014^1^** | **% in 2014** | **Indigenous Peoples in 2025** | **% in publications (latest 2025)** | **Difference between 2014 and 2025** |
| --- | --- | --- | --- | --- | --- |
| Argentina | 955,000 | 2.4% | 1,306,730 | 2.9% ^2^ | 351,730 |
| Bolivia | 6,200,000 | 62.2% | 4,302,484 | 38.7% ^3^ | -1,897,516 |
| Brazil | 900,000 | 0.5% | 1,693,535 | 0.8% ^4^ | 793,535 |
| Chile | 1,800,000 | 11.0% | 2,185,792 | 12.8% ^5^ | 385,792 |
| Colombia | 1,600,000 | 3.4% | 1,500,000 | 3.4% ^6^ | -100,000 |
| Costa Rica | 105,000 | 2.4% | 100,000 | 2.4% ^7^ | -5,000 |
| Ecuador | 1,000,000 | 7.0% | 1,301,887 | 7.7% ^8^ | 301,887 |
| El Salvador | 14,500 | 0.2% | 68,148 | 1.1% ^9^ | 53,648 |
| French Guiana | Not reported | Not reported | 10,000 | 4% ^10^ |  |
| Guatemala | 5,900,000 | 41.0% | 7,700,000 | 43.8% ^7^ | 1,800,000 |
| Honduras | 537,000 | 7.0% | 601,824 | 7.3% ^11^ | 64,824 |
| Mexico | 17,000,000 | 15.1% | 23,200,000 | 18.4% ^7^ | 6,200,000 |
| Nicaragua | 520,000 | 8.9% | 566,500 | 10.0% ^7^ | 46,500 |
| Panama | 420,000 | 12.3% | 698,114 | 17.2% ^7^ | 278,114 |
| Paraguay | 113,000 | 1.8% | 140,206 | 2.3% ^12^ | 27,206 |
| Peru | 7,000,000 | 24.0% | 5,972,603 | 26.0% ^13^ | -1,027,397 |
| Uruguay | 77,000 | 2.4% | 223,000.0 | 6.4% ^14^ | 146,000 |
| Venezuela | 725,000 | 2.7% | 724,592.0 | 2.8% ^15^ | -408 |

References

1. Commission for Latin America and the Caribbean (CEPAL). Indigenous Peoples in Latin America [Internet]. Work area: Population and development – Indigenous peoples and Afro-descendants. Santiago (Chile): CEPAL; 2014 Sep 22 [cited 2025 Sep 2]. Available from: https://www.cepal.org/en/infographics/indigenous-peoples-latin-america
2. International Work Group for Indigenous Affairs (IWGIA). Argentina – Indigenous World 2025. Copenhagen: IWGIA; 2025. Available from: [https://iwgia.org/es/argentina/5739-mi-2025-argentina.html]
3. Urgente.bo. Baja el número de bolivianos que se autoidentifican como indígenas: de 62 % en 2001 a 38,7 % en 2024 [Internet]. Urgente.bo; 2025 Aug 28 [cited 2025 Sep 3]. Available from: https://www.urgente.bo/noticia/baja-el-n%C3%BAmero-de-bolivianos-que-se-autoidentifican-como-ind%C3%ADgenas-de-62-en-2001-387-en-2024
4. International Work Group for Indigenous Affairs (IWGIA). Brazil – Indigenous World 2024. Copenhagen: IWGIA; 2024. Available from: [https://iwgia.org/en/brazil/5378-iw-2024-brazil.html]
5. International Work Group for Indigenous Affairs (IWGIA). Chile – El Mundo Indígena 2025. Copenhagen: IWGIA; 2025. Available from: [https://iwgia.org/es/chile/5729-mi-2025-chile.html]
6. International Work Group for Indigenous Affairs (IWGIA). Colombia. Copenhagen: IWGIA; n.d. Available from: [https://iwgia.org/es/colombia.html]
7. International Work Group for Indigenous Affairs (IWGIA). The Indigenous World 2025. Copenhagen: IWGIA; 2025. Available from: [International Work Group for Indigenous Affairs (IWGIA). The Indigenous World 2025. Copenhagen: IWGIA; 2025. Available from: [https://iwgia.org/doclink/iwgia-the-indigenous-world-2025-eng-online/eyJ0eXAiOiJKV1QiLCJhbGciOiJIUzI1NiJ9.eyJzdWIiOiJpd2dpYS10aGUtaW5kaWdlbm91cy13b3JsZC0yMDI1LWVuZy1vbmxpbmUiLCJpYXQiOjE3NDU2MDk2MDQsImV4cCI6MTc0NTY5NjAwNH0.hgvJa9yhC\_tm7rH3QUg5awj7j468f2mbhOhNbVRiQUU]
8. International Work Group for Indigenous Affairs (IWGIA). Ecuador – El Mundo Indígena 2025. Copenhagen: IWGIA; 2025. Available from: [https://iwgia.org/es/ecuador/5733-mi-2025-ecuador.html]
9. Wikipedia contributors. Censo salvadoreño de 2024. In: Wikipedia \[Internet]. San Francisco (CA): Wikimedia Foundation; 2024. Available from: [https://es.wikipedia.org/wiki/Censo\_salvadore%C3%B1o\_de\_2024]
10. International Work Group for Indigenous Affairs (IWGIA). French Guiana. Copenhagen: IWGIA; n.d. Available from: [https://iwgia.org/en/french-guiana.html]
11. Wikipedia contributors. Grupos étnicos de Honduras. In: Wikipedia \[Internet]. San Francisco (CA): Wikimedia Foundation; n.d. Available from: [https://es.wikipedia.org/wiki/Grupos\_%C3%A9tnicos\_de\_Honduras]
12. International Work Group for Indigenous Affairs (IWGIA). Paraguay – Indigenous World 2025. Copenhagen: IWGIA; 2025. Available from: [https://iwgia.org/en/paraguay/5748-iw-2025-paraguay.html]
13. International Work Group for Indigenous Affairs (IWGIA). Perú – El Mundo Indígena 2025. Copenhagen: IWGIA; 2025. Available from: [https://iwgia.org/es/peru/5751-mi-2025-per]
14. Newsroom Infobae. La población de Uruguay crece 2,5 % y llega a casi 3,5 millones de habitantes, según censo [Internet]. Montevideo (Uruguay): Infobae; 2025 Feb 23 [cited 2025 Sep 3]. Available from: https://www.infobae.com/america/agencias/2024/12/10/la-poblacion-de-uruguay-crece-25-y-llega-a-casi-35-millones-de-habitantes-segun-censo/
15. International Work Group for Indigenous Affairs (IWGIA). Venezuela – El Mundo Indígena 2024. Copenhagen: IWGIA; 2024. Available from: [https://iwgia.org/es/venezuela/5491-mi-2024-venezuela.html]
